# Gene flow from weedy rice to T1c-19 transgenic rice stacked with cry1C*/bar genes and fitness of F_1_ hybrids

**DOI:** 10.3389/fpls.2025.1513367

**Published:** 2025-07-22

**Authors:** Guang-Le Xie, Jia-Qi Shen, Min Wang, Ji-Kun Li, Yao Huang, Sheng Qiang, Xiao-Ling Song, Wei-Min Dai

**Affiliations:** Weed Research Laboratory, College of Life Sciences, Nanjing Agricultural University, Nanjing, China

**Keywords:** transgenes, herbicide-resistant rice, T1c-19, weedy rice, reverse gene flow, reverse F_1_ hybrids, fitness

## Abstract

**Introduction:**

Bidirectional gene flow via pollen between transgenic rice and weedy rice could occur in natural fields. Gene flow from transgenic rice to weedy rice has been confirmed in many studies, and thus results showed that F_1_ hybrids could persist in natural agroecosystems due to their unimpaired reproductive ability. However, the reverse gene flow from weedy rice to transgenic rice is rarely reported.

**Method:**

We quantified reverse gene flow from three weedy rice accessions to transgenic rice line T1c-19 with cry1C*/bar. In field trials with alternating layout of cultivating transgenic rice and weedy rice accessions and adjacent layout cultivating them in a close vicinity, the reverse gene flow was detected. And the fitness of reverse F_1_ (RF_1_) hybrids obtained by manual pollination using T1c-19 as maternal plants and weedy rice as paternal plants was evaluated in field.

**Result:**

No gene flow from WRTZ was observed, while gene flows from WRMM were observed at 0.0508% and 0.0808%, respectively, and those from WRYY were 0.0692% and 0.1008%, respectively. RF_1_ plants exhibited significantly higher composite fitness compared to their weedy rice counterparts, due to enhanced fecundity-related traits observed under both insect pressure and no-insect pressure conditions. However, the impact of reverse gene flow may be limited because RF_1_ hybrid seeds presented lower seed shattering, and therefore most of it would be harvested by combine harvester.

**Discussion:**

Our study revealed that gene flow from three weedy rice accessions to T1c-19 could produce RF_1_ hybrids with greater composite fitness. Any loss of seeds into agroecosystem may result in a greater risk of RF1 hybrids due to their morphological similarity and high fitness.

## 1 Introduction

Rice (*Oryza sativa* L.) is a worldwide food crop that plays a pivotal role in China’s food production. A large number herbicide resistant (HR), insect resistant (IR), even stacked traits rice lines have been bred by transgenic or mutation technique and the HR rice by mutation technique have been planted in China (Tang et al., 2006; Chen et al., 2021). While the commercial release of HR rice will bring economic and social benefits, it also poses certain ecological risks (Nicolia et al., 2014; Merotto et al., 2016; Clark and Maselko, 2020). One of the major concerns in relation to the ecological risks is HR gene flow via pollen to its weedy relative weedy rice (*Oryza sativa* L.) (Messeguer, 2003; Sun et al., 2015; Nam et al., 2019). Once the HR gene flows into weedy rice, hybrids carrying the HR gene could survive under herbicide selection, making the control of weedy rice more challenging. Meanwhile, HR gene may persist and spread within the weedy rice population by both gene flow via pollen and seed dispersal. This could result in an aggravated weedy rice problem that, combined with the technical difficulties of control, could jeopardize the HR rice technology itself. This has been the case with non-transgenic imidazolinone resistant rice (IMR-rice, Clearfield®) (Rajguru et al., 2005; Roso et al., 2010; Busconi et al., 2012; Merotto et al., 2016; Avila et al., 2021; Unan et al., 2024). Thus, HR weedy rice may become a significant barrier to continued HR cultivated rice (Messeguer et al., 2004; Busconi et al., 2014; Merotto et al., 2016; Dauer et al., 2018).

Weedy rice is present in most rice-growing areas worldwide (Arrieta-Espinoza et al., 2005; Olsen et al., 2007; Shivrain et al., 2009; Karn et al., 2020). It has widely evolved from cultivated rice via de-domestication (Sun et al., 2019; Qiu et al., 2017, 2020; Wu et al., 2021). Wild rice (*Oryza rufipogon*) hybridization with weedy rice has contributed substantially to the evolution of Southeast Asian weedy rice, with some strains acquiring weed-adaptive traits through introgression from the wild progenitor (Li et al., 2024). Weedy rice has strong feral characteristics including robust seed shattering and dormancy, seedling vigor, early maturity, and greater competitive advantage, which are critical for its ability to survive and persist in rice fields (Thurber et al., 2011; Dai et al., 2014, 2017; Zhao et al., 2018, 2020, 2021). It has become the primary weed-related factor constraining increased rice yields in China, including Jiangsu, Heilongjiang, Ningxia and Guangdong to the whole East China, Northeast China, Northwest China and South China, causing increasing losses to rice production (Ma et al., 2005; Wang et al., 2023; Cai et al., 2023).

Whether the HR gene can successfully flow to weedy relatives and pose a threat to agricultural production and the environment depends on the rate at which the transgenic crop can hybridize with weedy relatives and, particularly, on the fitness of the hybrids (Song et al., 2011; Zuo et al., 2011; Liu et al., 2016; Dauer et al., 2018; Huang et al., 2019). Fitness is the ability of an individual to survive and reproduce under specific environmental conditions. It determines whether hybrids can survive and establish populations in nature (Jenczewski et al., 2003; Lu and Yang, 2009; Xia et al., 2011). Numerous studies have determined that the fitness of hybrids carrying a HR gene is closely related to several factors, including parental genetic background, and environmental conditions (with or without various selection pressures, competition) (Cao et al., 2009; Vila-Aiub et al., 2009; Song et al., 2011; Yang et al., 2011; Darmency et al., 2015; Vila-Aiub, 2019).

The HR gene flow via pollen from cultivated rice to weedy rice has been confirmed in many studies, with an outcrossing rate usually less than 1% (Zhang et al., 2003; Messeguer, 2003; Chen et al., 2004; Zhang et al., 2006; Shivrain et al., 2007, 2008; Zuo et al., 2011; Sun et al., 2015; Nam et al., 2019). F_1_ hybrids frequently exhibit heterosis for vegetative traits but have lower reproductive capacity than the corresponding weedy rice parent because of their lower seed set (Zhang et al., 2008; Sanchez-Olguin et al., 2009). However, some F_1_ hybrids display similar seed set as well as composite agronomic performance (Song et al., 2011; Liu et al., 2016) or produced significantly more and heavier grains (Nam et al., 2019). For insect-resistant transgenic rice and weedy rice, hybrids under low insect pressure exhibited fitness cost, and fitness advantage under high insect pressure (Cao et al., 2009; Yang et al., 2011). These researchers suggested that the HR or IR F_1_ hybrids could persist under natural agroecosystem because these F_1_ hybrids presented certain reproductive ability even unimpaired one.

Gene flow between HR cultivated rice and weedy rice is bidirectional; direct gene flow occurs from cultivated to weedy rice, while indirect or reverse gene flow occurs in the opposite direction. Reverse gene flow under field conditions could result in the transfer of dominant weedy traits to HR cultivars, potentially leading to the emergence of HR weedy rice (Shivrain et al., 2009; Serrat et al., 2013; Zhang et al., 2018).

This reverse gene flow might significantly impact the evolutionary dynamics of weedy rice populations. The segregating progeny of HR transgenic hybrid rice pollinated by weedy rice rapidly develop into weedy forms carrying the HR transgene under natural conditions (Zhang et al., 2018). Although bidirectional gene flow between HR cultivated rice and weedy rice can occur and produce HR weedy rice, reverse gene flow has received less attention compared to direct gene flow from HR cultivated rice to weedy rice. One of the main reasons is the more complex methods required to verify reverse F_1_ hybrids (RF_1_) produced by reverse gene flow, compared to F_1_ hybrids resulting from direct gene flow, which can be tested by herbicide application or PCR analysis for the HR gene.

Reverse gene flow from imidazolinone-resistant rice (IMI-R; Clearfield® rice) to red rice has been documented in two cases (Shivrain et al., 2009; Serrat et al., 2013). The initially selected putative reverse flow (PRF) seedlings were identified based on their most important phenotypic characteristic—vigorous growth, characterized by faster and taller growth. PRF plants were then analyzed using the *Oryza* simple sequence repeat (SSR) primers RM234 and RM253 or by determining their molecular fingerprint patterns through AFLP analysis. These studies supported the possibility that HR weedy rice could arise through reverse gene flow from weedy rice to HR rice.

Red pericarp is a typical and convergent characteristic of weedy rice (Sun et al., 2019; Qiu et al., 2020). The *Rc* gene, located on rice chromosome 7, contains eight exons and confers a red pericarp. The *Rc* allele *rc*, found in most white pericarp rice genotypes, is characterized by the absence of a 14 bp fragment in the seventh exon (Sweeney et al., 2007; Li et al., 2014). If gene flow occurred from weedy rice to HR rice, both fragments of 118 bp and 104 bp should be present in RF_1_ hybrids (Huang et al., 2019). Therefore, both alleles can serve as markers for detecting reverse gene flow.

The insect-resistant and glufosinate-resistant stacked transgenic rice line T1c-19 was successfully developed by Huazhong Agricultural University (Tang et al., 2006). The weediness of T1c-19 under no selection pressure, gene flow from T1c-19 to weedy rice, and the fitness of its F_1_ hybrids have been reported (Huang et al., 2015; 2016; 2019). T1c-19 did not exhibit weedy characteristics; it had weak overwintering ability, low seed shattering, and failed to establish volunteer plants. The gene flow from T1c-19 rice to weedy rice ranged from 0.230% to 0.106%, depending on the genotype of the weedy rice and field planting design. Compared to their weedy rice counterparts, the F_1_ hybrids displayed either higher performance or non-significant changes in the presence or absence of insect pressure in mixed planting with weedy rice. The F_1_ hybrids also showed non-significant changes regardless of glufosinate pressure, insect pressure, in monoculture planting. The potential risk of gene flow from T1c-19 to weedy rice should be considered due to the greater fitness advantage of F_1_ hybrids in most cases.

However, reverse gene flow from weedy rice to T1c-19 and the fitness of reverse F_1_ hybrids (RF_1_) using weedy rice as the pollen donor have not been reported. In this study, the method for verifying the RF_1_ hybrids was established based on the *Rc* gene. A field experiment was conducted to assess the reverse gene flow from three weedy rice accessions to T1c-19 and to compare the fitness of the RF_1_ hybrids, under no insect and natural insect selection pressures, with their paternal weedy rice counterparts. The results from this study should be useful for regulatory authorities to consider reverse gene flow and develop relevant policies for assessing the risks associated with bidirectional gene flow. These findings are also helpful for regulatory authorities in making decisions regarding the authorization of T1c-19. Furthermore, the data provide an experimental basis for developing risk assessment standards for stacked-trait rice.

## 2 Materials and methods

### 2.1 Plant materials

The insect-resistant and glufosinate-resistant stacked transgenic rice line T1c-19 contains two tightly linked genes: the cry1C* insect resistance gene and the *bar* glufosinate resistant gene. This line was generated through *Agrobacterium*-mediated co-transformation of the indica rice (*O. sativa* ssp. *indica*) cultivar MH63, an elite cytoplasmic male-sterile (CMS) *restorer* line (Tang et al., 2006). T1c-19 was provided by the State Key Laboratory of Crop Genetic Improvement, Huazhong Agricultural University, China.

Three weedy rice accessions, WRMM, WRTZ, and WRYY, were selected from the germplasm collection of the Weed Research Laboratory at Nanjing Agricultural University (NJAU), China (**Table 1**).

**Table 1:** Origin and key characteristics of weedy rice accessions used in the experiment.

Accession line	Accession code (for weedy rice)	Origin Locality	Subspecies	Pericarp color	Awn
WRMM	WRGD008	Maoming, Guangdong Province	Indica	Red	Absent
WRTZ	WRJS015	Taizhou, Jiangsu Province	Indica	Brown	Absent
WRYY	WRHU011	Yiyang, Hunan Province	Indica	Red	Absent

The F_1_ hybrids obtained by manual pollination between weedy rice (as the paternal parent) and T1c-19 (as the maternal parent) were designated as RF_1_ to distinguish from the F_1_ hybrids obtained by manual pollination between weedy rice (as maternal parent) and T1c-19 (as paternal parent) (**Table 2**). All RF_1_ plants were transgene positive. Since three weedy rice accessions were used as paternal parents, three different RF_1_ hybrids were obtained by hand pollination in 2014 and further identified by the site of origin of the male parent (**Table 2**). Mature seeds were harvested, dried at 25 °C, and stored at 4°C until use.

**Table 2:** Types and characteristics of RF_1_ hybrids used in the experiments.

Types	Hybrids (♀×♂)	Abbreviation
F_1_	F_1_(T1c-19×WRMM)	RF_1_MM
	F_1_(T1c-19×WRTZ)	RF_1_TZ
	F_1_(T1c-19×WRYY)	RF_1_YY

### 2.2 Methods

#### 2.2.1 Gene flow from weedy rice to T1c-19

##### 2.2.1.1 Arrangement of field experiment

To detect the maximum possible gene flow frequency from weedy rice to T1c-19, two different experimental designs, alternating and adjacent cultivation, were conducted at the Jiangpu Experimental Station (N32.011569 E118.624535), part of the Weed Research Laboratory of Nanjing Agricultural University, China. The experimental fields, where the studies were conducted from May to October in 2016, were authorized by Ministry of Agriculture and Rural Affairs of the People’s Republic of China (MOA). The experimental area was surrounded by a 100 m wide corn crop, providing a buffer zone without rice crop. All rice cultivation practices, including irrigation, fertilization, and pest management followed those common in rice production in China. Weed control was performed manually as needed.

T1c-19 was sown on 18^th^ June, three days earlier than both weedy rice sown on 21^st^ June for the alternating cultivation design trial and the first batch weedy rice in adjacent cultivation design trial. This adjustment was necessary because T1c-19 flowers three days earlier than three weedy rice accessions, according to our preliminary experiments. The second batch weedy rice seeds in the adjacent cultivation design trial were sown on 26^th^ June (five days after the first batch) to ensure flowering synchronization with the pollen recipient T1c-19. One-month-old seedlings were transplanted according to the following experimental designs. Each experiment involving a weedy rice accession was conducted in field using a complete randomized block design with four replications.

##### 2.2.1.2 Alternating cultivation design trial

**Figure 1:**
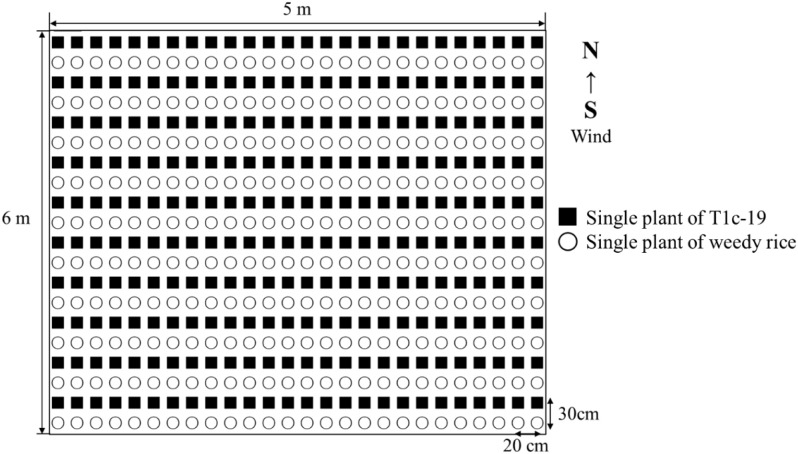
Layout of an individual plot in the alternating cultivation for gene flow from weedy rice (○) to T1c-19 (■).

The seedlings of each weedy rice accession and T1c-19 were transplanted into individual 5×6 m plots in alternating rows, planted at a density of one seedling per hole. The inter-row and interplant distances were 30 cm and 20 cm, respectively (**Figure 1**). The rows were aligned with the prevailing wind direction (from South to North). Each plot contained 20 rows, with 26 seedlings per row. Plots were separated by a distance of 200 cm and each plot was replicated four times resulting in a total of 12 experimental plots. This design mitigated potential biases in gene flow due to the equal distance between each transgenic and weedy rice plant.

##### 2.2.1.3 Adjacent cultivation design trial

**Figure 2:**
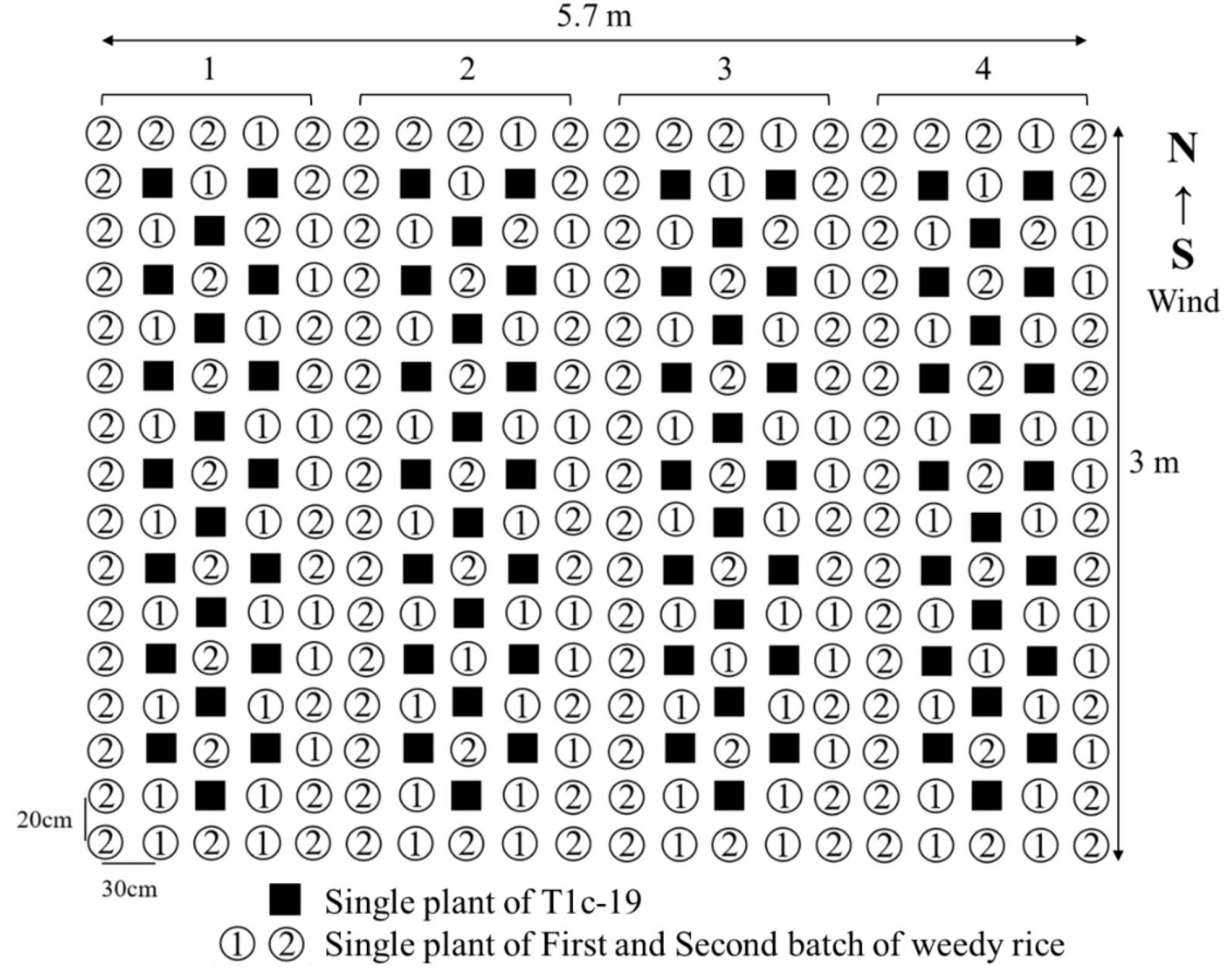
Layout of an individual plot in the adjacent cultivation for gene flow from two batches weedy rice (① and ②) to T1c-19 (■).

The seedlings of each weedy rice accession and T1c-19 were transplanted into individual 5.7×3.0 m plots, including four blocks (**Figure 2**). The seedlings were manually transplanted at a spacing of 30 cm between rows and 20 cm between plants. Each plot contained 84 seedlings of T1c-19, with 96 and 140 seedlings in the first and second batches of weedy rice, respectively. Plots were separated from each other by 60 cm. This experiment was conducted using a complete randomized block design with four replications, for a total of 12 experimental plots.

##### 2.2.1.4 Field data and seed collection

Plant height of both T1c-19 and weedy rice individuals was measured at the end of the flowering period. The dates of first, peak, and last flowering were recorded to estimate the days of flowering overlap between the pollen donor and recipient plants. The first (approximately 5% of total flowers per panicle opening in one day), peak (approximately 30–80% of total flowers per panicle opening in one day) and the final flowering time (approximately 95% of total flowers per panicle opening in one day) was observed in the adjacent cultivation experiment according to Sun et al. (2015).

Seeds from the pollen recipient T1c-19 in the two experiments were harvested in October and thoroughly mixed for each replication, in order to ensure reliability for testing gene flow frequency. To calculate gene flow frequency, approximately 30,000 seeds were randomly selected from each replication, which is the number of seeds required for testing gene flow frequency if it exceeds 0.05% (Wang et al., 2020).

##### 2.2.1.5 Procedures to verify reverse gene flow

The three weedy rice accessions possess the *Rc* gene that confers a red pericarp, while T1c-19 has the known 14-bp deletion in the *Rc* gene that confers the white pericarp characteristic of cultivated rice. To enhance the efficiency of testing the *Rc* gene in seeds collected from pollen recipients T1c-19, an optimized method for detecting hybrids produced via pollen from weedy rice to T1c-19 was established (**Supplementary File**). Sixty seeds, randomly selected from those collected from T1c-19 within the same plot, were grouped into a total of 500 groups, amounting to 30,000 seeds. These seeds were first dehulled, and each group seeds were ground completely to pass through a 60-mesh sieve. Subsequently, genomic DNA was extracted from ground tissue using the DP305 DNA Miniprep Kit produced by Tiangen Company (Shanghai, China). The method for the PCR analysis of the *Rc* gene followed that reported by Huang et al. (2019).

#### 2.2.2 Fitness of RF_1_ hybrids

All RF_1_ hybrids were obtained by hand pollination in 2014 using T1c-19 as the maternal parent and three weedy rice accessions as the paternal parent. The fitness of RF_1_ hybrids obtained in 2014 was evaluated in the same manner as the fitness of F_1_ in that year (Huang et al., 2019). Field trials were conducted at the Jiangpu Experimental Station (32.011569°N, 118.624535°E) of the Weed Research Laboratory of NJAU. The experimental studies were conducted from May to November 2014 and were authorized by the MOA. The experimental area was isolated by a 100-m-wide corn crop. Pregerminated seeds of hybrids were sown in seedling trays (54 cm×29 cm×5 cm) filled with field soil at a density of approximately 1400 individuals per m^2^.

Both expected amplification fragments of the *Rc* gene (118 bp, and 104 bp) should be present in true F_1_ hybrids, with T1c-19 as maternal plants and the three weedy rice accessions as paternal parents. At the four to five leaf stage, all RF_1_ hybrids were tested for the *Rc* gene. T1c-19 and WRMM accessions were used as positive and negative controls, respectively. The methods for testing the *Rc* gene in RF_1_ were the same as those described by Huang et al. (2019).

##### 2.2.2.1 Variables measured in the field

At the five–six leaf stage, selected uniform RF_1_ plants were transplanted into the experimental field. The plot experiments included two forms of selection pressures: with and without natural insect pressure. Under monoculture planting, plots contained 36 individuals in a 6×6 grid with a 20-cm spacing.

In plots with natural insect pressure, plants were not treated with insecticides or any other insect control measures. Absence of natural insect pressure was achieved by covering the experimental plots with an anti-insect net and supplementing this with insecticide application as described as Huang et al. (2019).

Insect damage was assessed by counting the blasted tillers and folded leaves at the heading stage within the weedy rice plants. An insect index was calculated as follows: Insect index (%)=Leaf folded (%)+Tillers blasted (%) (Xia et al., 2011).

For each plant type, 10 individuals per plot were randomly selected at maturity for measuring fitness variables, excluding for those in the border rows to avoid edge effects. All fitness variables were measured according to the methods described by Song et al. (2011). These variables included plant height, effective panicle number per plant, filled grain number per panicle, yield per plant, 1000-grain weight and seed set.

##### 2.2.2.2 Statistical analysis

The mean plant height of T1c-19 and weedy rice was calculated in the gene flow experiment. One-way ANOVA (Dunnett’s Multiple Range Test, DMRT) was used to examine significant differences in plant height between T1c-19 and two batches of weedy rice in the adjacent cultivation. The t-test was used to examine significant differences in plant height between T1c-19 and weedy rice in the adjacent cultivation experiment.

Composite fitness across the six fitness-related traits measured from vegetative to mature stages was calculated using the methods outlined for F_1_ by Huang et al. (2019).

One-way ANOVA was used to examine differences in the insect index among the hybrids and the respective weedy rice accessions under natural insect pressure. The means of each trait and composite fitness for the hybrids and their respective weedy rice accessions, along with the values for composite fitness of hybrids, were compared using the t-test for independent samples in SPSS version 17 (SPSS Inc., 2008).

Given that the fitness of RF_1_ and F_1_ was assessed in the same year under identical experimental conditions, the relative fitness of RF_1_/F_1_ was compared using the same methodology as that used RF_1_ and their weedy rice counterparts.

## 3 Results

### 3.1 Gene flow from weedy rice to T1c-19

#### 3.1.1 Flowering period

Due to being sown and transplanted on the same day in both the alternating the adjacent experiments, T1c-19 began flowering on 22 September, with a flowering duration of 20 days in both experiments. The flowering overlap between T1c-19 and WRMM, WRYY and WRTZ in the alternating experiment was 5, –6 and –8 days, respectively (**Table 3**). In adjacent experiment, the overlap between T1c-19 and WRMM, WRYY and WRTZ in the first and second batches was 5, 6, and 8 days, and 6, 8 and 11 days, respectively (**Table 4**).

**Table 3:** Flowering and overlaps periods of weedy rice with T1c-19 in alternation cultivation experiment for gene flow from weedy rice to T1c-19.

Accession	Date (date/month)					Overlap (d)
	Sowing	Transplanting	First flowering	Peak flowering	Final flowering	
T1C-19	18, June	21, July	22, Sep.	27, Sep.	11, Oct.	—
WRMM	21, June	21, July	13, Sep.	20, Sep.	26, Sep.	5
WRYY	21, June	21, July	17, Sep.	22, Sep.	27, Sep.	6
WRTZ	21, June	21, July	17, Sep.	25, Sep.	29, Sep.	8

**Table 4:** Flowering periods and overlaps of weedy rice accessions with T1c-19 in adjacent cultivation experiment for gene flow from weedy rice to T1c-19.

Accession	Date (date/month)					Overlap (d)
	Sowing	Transplanting	First flowering	Peak flowering	Final flowering	
T1C-19	18, June	21, July	22, Sep.	27, Sep.	11, Oct.	—
WRMM-1	21, June	21, July	13, Sep.	20, Sep.	26, Sep.	5
WRMM-2	26, June	26, July	16, Sep.	21, Sep.	27, Sep.	6
WRYY-1	21, June	21, July	17, Sep.	22, Sep.	27, Sep.	6
WRYY-2	26, June	26, July	19, Sep.	22, Sep.	29, Sep.	8
WRTZ-1	21, June	21, July	14, Sep.	25, Sep.	29, Sep.	8
WRTZ-2	26, June	26, July	18, Sep.	28, Sep.	2, Oct.	11

*-1 and -2 refers to the planting timing, first batch and the second batch of weedy rice.

#### 3.1.2 Flowering time

**Figure 3:**
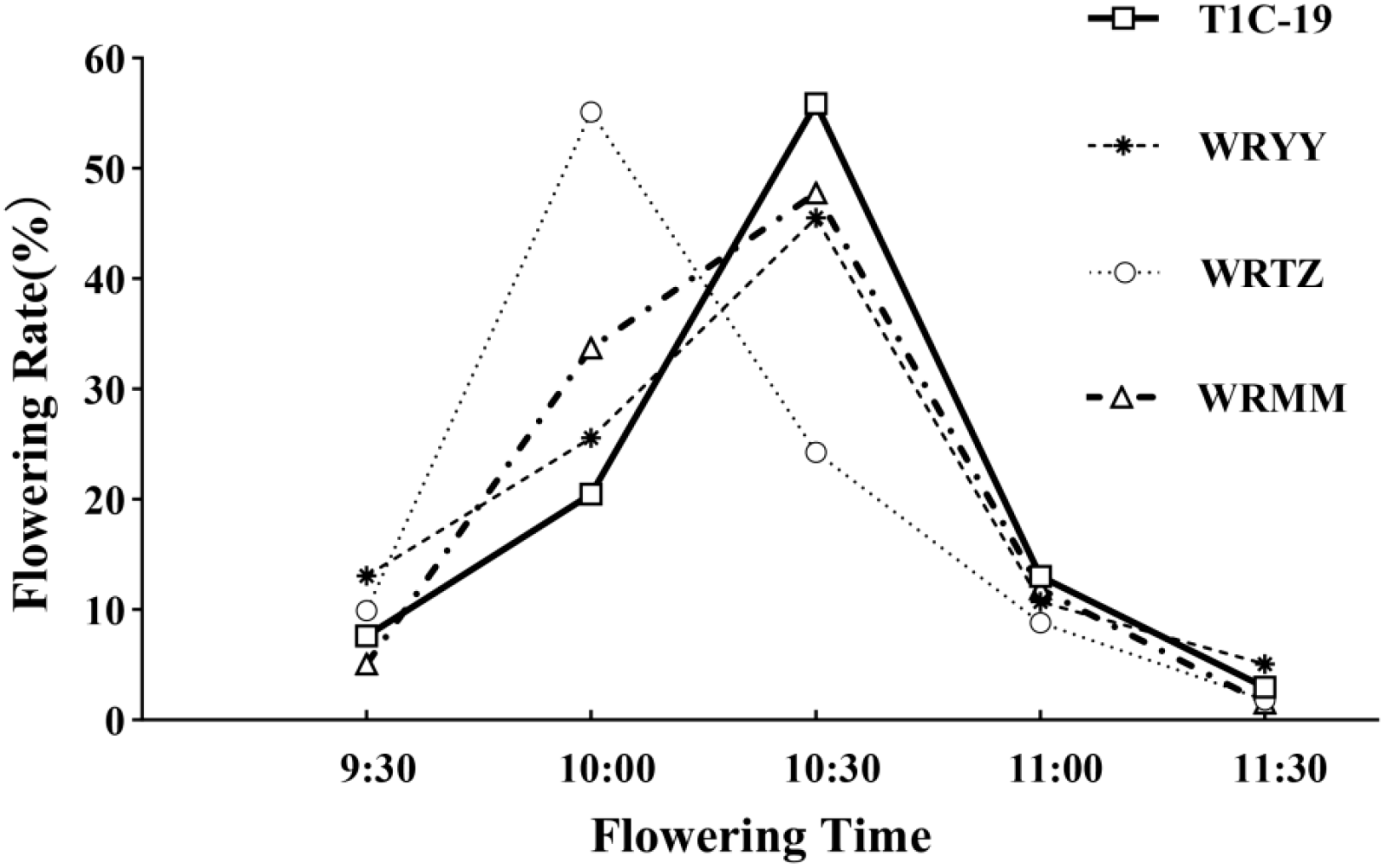
Daily flowering rhythm of weedy rice in relation to T1c-19 in alternation and adjacent cultivation experiments for gene flow from weedy rice to T1c-19.

In both the alternating and adjacent cultivation, T1c-19 and the three weedy rice accessions had similar flowering patterns (**Figure 3**). T1c-19 began flowering at 09:30, reached peak flowering at 10:30, and ceased flowering by 11:30. The daily flowering rhythms of WRMM and WRYY were consistent with that of T1c-19. WRTZ began and ceased flowering synchronously with T1c-19 but peaked at 10:00, which was 30 min earlier than T1c-19. The flowering time period of T1c-19 overlapped with the three weedy rice by approximately two hours.

#### 3.1.3 Plant height

**Figure 4:**
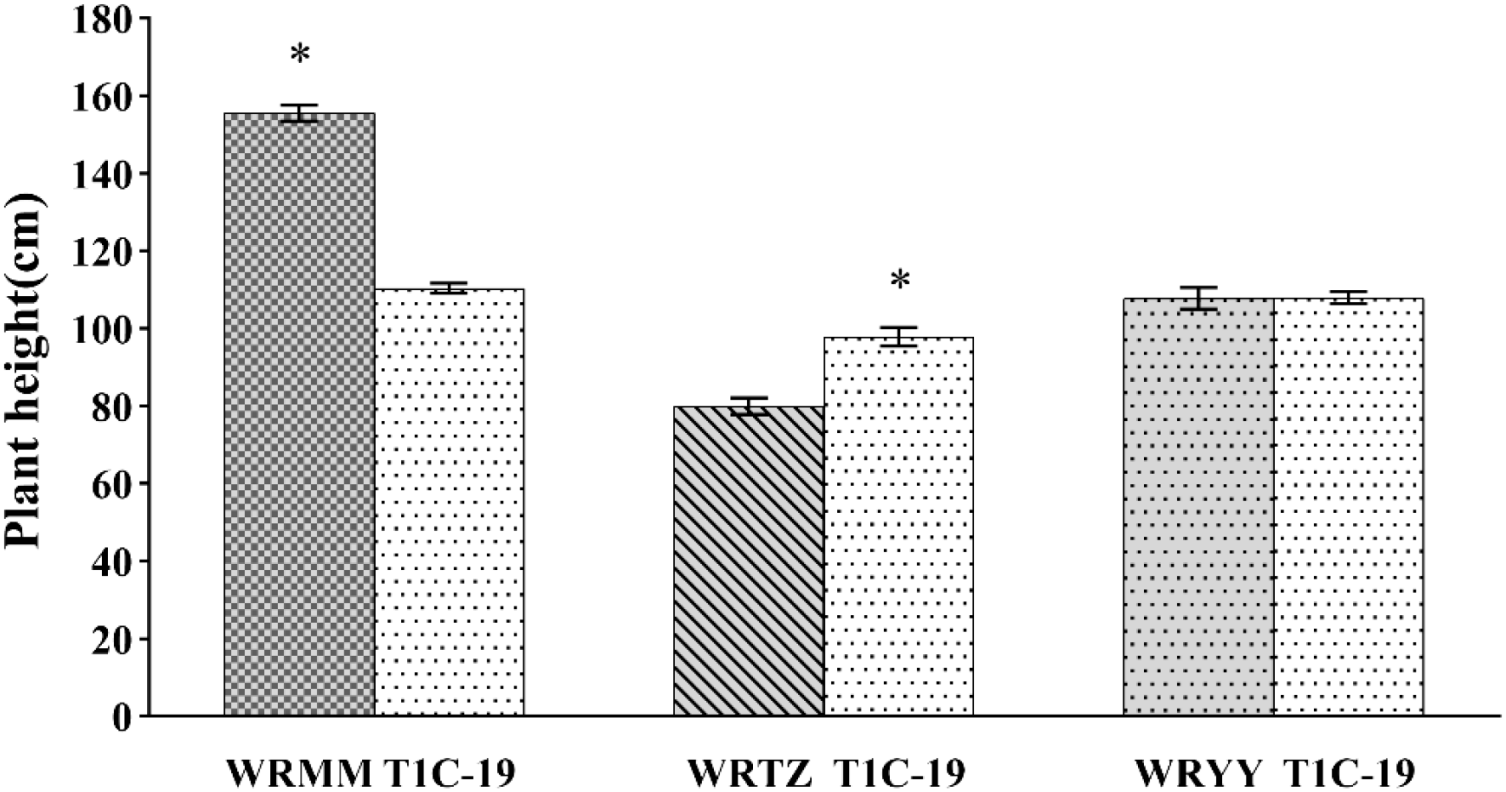
Plant height (mean ± SE) of weedy rice with T1c-19 in the alternating cultivation experiment for gene flow from weedy rice to T1c-19. * means significant difference at 0.05 between weedy rice and T1c-19 by using t-test.

In the alternating experiment, the plant heights of T1c-19 with WRMM, WRTZ and WRYY were 110.4 cm、97.9 cm、108 cm, respectively. The height of T1c-19 was significantly lower by 45.20 cm compared to WRMM, 17.90 cm higher than WRTZ, and similar to WRYY (**Figure 4**).

**Figure 5:**
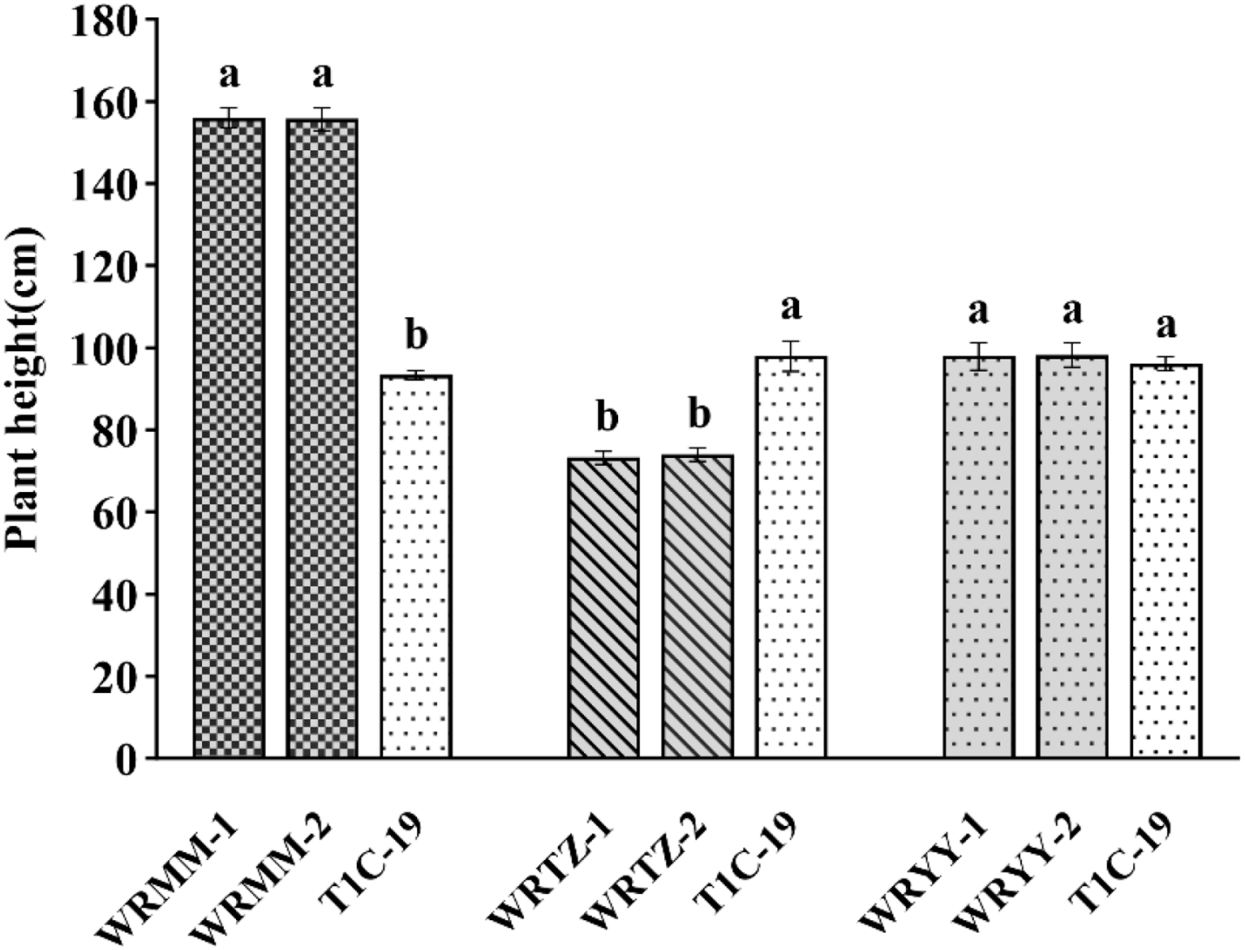
Plant height (mean ± SE) of weedy rice with T1c-19 in the alternating adjacent cultivation experiment for gene flow from weedy rice to T1c-19. The same letters indicate no significant difference at 0.05% by DMRT.

In the adjacent experiment, T1c-19 was significantly shorter by 62 cm than WRMM-1 and WRMM-2, 24.8 and 24 cm taller than WRTZ-1 and WRTZ-2, and similar in height to WRYY (**Figure 5**).

#### 3.1.4 Verification of gene flow

**Figure 6:**
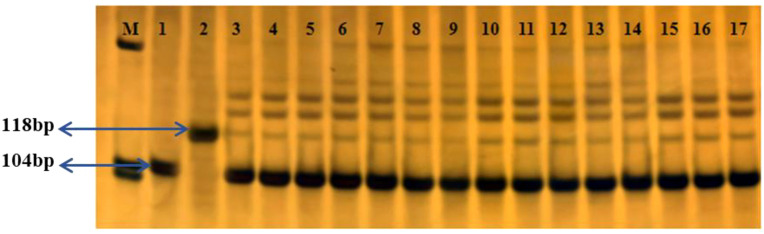
The gene *Rc* Polymerase chain reaction (PCR) for the detection of the *Rc* gene in seeds of T1c-19 in the alternating and adjacent cultivation experiments for gene flow from weedy rice to T1c-19. M: Marker; 1: transgenic rice T1c-19; 2: WRYY; 3-6: T1c-19 and WRMM in the alternating cultivation; 7-10: T1c-19 and WRYY in the alternating cultivation; 11-14: T1c-19 and WRMM in the adjacent cultivation; 15-17: T1c-19 and WRYY in the adjacent cultivation.

The 118 bp *Rc* gene fragment, present in the weedy rice, was not detected in any of the 30,000 T1c-19 seeds collected from each plot in both the alternating and adjacent experiments with WRTZ. This indicates that gene flow from WRTZ to T1c-19 did not occur. For WRMM, out of 500 tests per plot, the 104 bp and 118 bp fragments were detected 13, 15, 16, and 17 times in the alternating experiment and 26, 22, 25, and 24 times in the adjacent experiment. The average gene flow frequency was 0.0508% in the alternating experiment and 0.0808% in the adjacent experiment. For WRYY, the fragments were detected 23, 21, 20, and 19 times in the alternating experiment and 29, 32, 33, and 27 times in the adjacent experiment, resulting in average gene flow frequency of 0.0692% and 0.1008%, respectively (**Figure 6**).

### 3.2 Fitness of RF_1_

#### 3.2.1 Verification of RF_1_ hybrids

**Figure 7:**
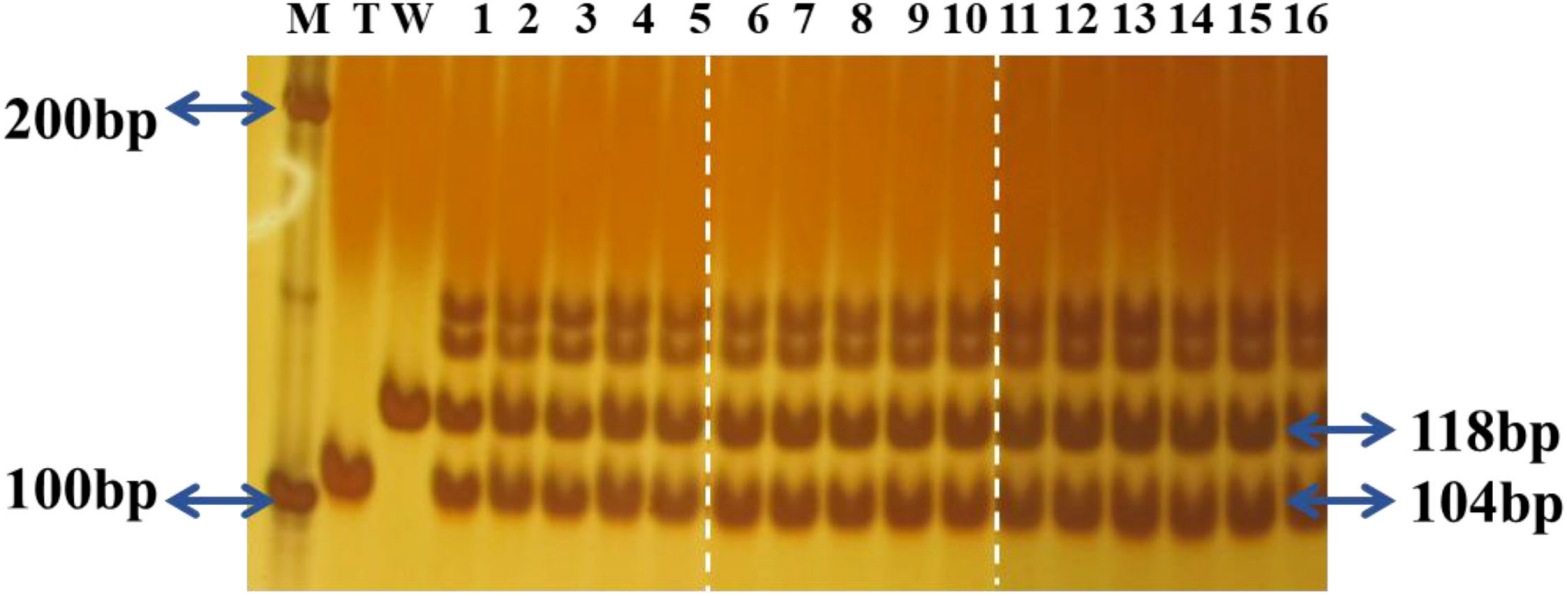
Polymerase chain reaction (PCR) detection of the *Rc* gene in F_1_ hybrids using T1c-19 as maternal plants in the experiment. M: Marker; T:T1c-19; W: WRMM; 1-5: RF_1_MM; 6-10: RF_1_TZ; 11-16: RF_1_YY.The reassembled figure presents different regions from the same gel, with white dotted lines indicating the cropping boundaries.

All tested RF_1_ hybrids using T1c-19 as the maternal plant, carried the expected 118 bp and 104 bp fragments (**Figure 7**). Therefore, all putative RF_1_ used in the experiment were confirmed as true RF_1_ hybrids.

#### 3.2.2 Target insect pressure

**Figure 8:**
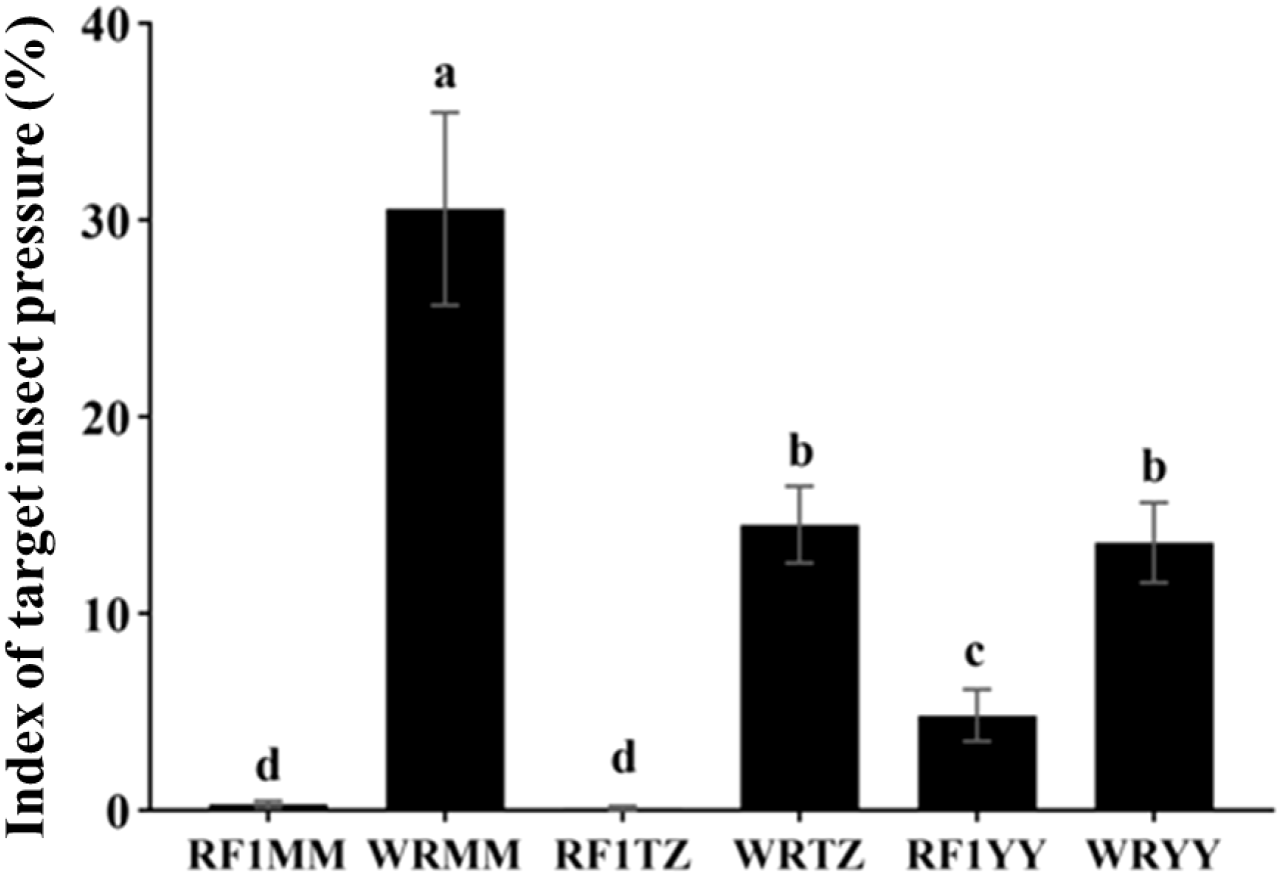
Index of target insect pressure under natural insect pressure in the monoculture planting. WRMM, WRTZ, and WRYY are weedy rice collected from Maoming, Taizhou and Yiyang, respectively. RF_1_MM, RF_1_TZ and RF_1_YY are RF_1_ hybrids of T1c-19 (as maternal plant) and weedy rice accessions (as paternal plants). Data are presented as mean ± SE (n=4). Different letters indicate significant differences among RF_1_ and weedy rice at P<0.05 by one-way ANOVA.

As expected, no target insect pressure was observed in the plots protected where insects were physically excluded. Under natural insect pressure, the target insect pressure on transgene-positive RF_1_ plants (average 1.75%) was significantly lower than on weedy rice (average 19.57%) (**Figure 8**).

#### 3.2.3 Fitness of RF_1_ and weedy rice

In the absence of insect pressure, compared to their respective weedy rice parents, RF_1_MM had approximately 21% greater 1000-grain weight, produced 34.6% more filled grains per panicle, and had 1.11 times more yield per plant compared to WRMM. RF_1_TZ exhibited superiority across all tested variables compared to WRTZ. RF_1_YY produced 30.4% more filled grains per panicle, 4.2% higher seed set, 27% greater 1000-grain weight, and nearly 1 time more yield per plant compared to WRYY (**Table 5**).

**Table 5:** Fitness-related traits of RF_1_ and weedy rice under non-insect pressure in pure planting.

Type	Plant height (cm)	Effective Panicle number/plant	Filled grain number/panicle	Seed set (%)	1000-grain weight (g)	Yield per plant (g)
RF_1_MM	143.78 ± 1.09	12.68 ± 0.45	124.00 ± 1.93*	88.07 ± 1.77	27.72 ± 0.08*	44.67 ± 1.82*
WRMM	150.56 ± 1.88	11.2 ± 0.70	92.13 ± 4.56	88.23 ± 1.51	22.9 ± 0.39	21.14 ± 0.76
RF_1_TZ	94.45 ± 2.65*	21.73 ± 0.79*	120.5 ± 3.58*	82.34 ± 0.16*	27.79 ± 0.33*	53.99 ± 3.7*
WRTZ	76.84 ± 1.96	19.15 ± 0.41	75.3 ± 2.18	75.8 ± 0.51	23.23 ± 0.84	23.23 ± 1.27
RF_1_YY	91.42 ± 0.33	12.7 ± 0.40	106.85 ± 3.82*	88.14 ± 0.78*	29.1 ± 0.44*	37.55 ± 1.00*
WRYY	88.38 ± 2.96	12.09 ± 0.50	81.91 ± 5.12	84.60 ± 0.70	22.91 ± 0.44	18.79 ± 0.46

*Means of each trait were separated using t-test for independent samples. *Significant differences between hybrids and their respective weedy rice at P<0.05 level. Data = Mean ± SE.

**Table 6:** Fitness-related traits of RF_1_ and weedy rice under natural insect pressure in pure planting.

Type	Plant height (cm)	Effective Panicle number/plant	Filled grain number/panicle	Seed set (%)	1000-grain weight (g)	Yield per plant (g)
RF_1_MM	146.6 ± 0.74	13.93 ± 0.3*	169.8 ± 3.66*	89.86 ± 0.68	27.84 ± 0.4*	48.43 ± 1.75*
WRMM	152.73 ± 0.88*	10.00 ± 0.70	117.27 ± 2.08	87.74 ± 1.91	21.47 ± 0.16	18.02 ± 1.97
RF_1_TZ	96.86 ± 1.54*	21.93 ± 1.38*	141.5 ± 4.87*	84.94 ± 1.4*	27.56 ± 0.84*	53.77 ± 2.95*
WRTZ	75.13 ± 4.06	12.65 ± 0.39	80.48 ± 8.34	73.80 ± 1.28	23.78 ± 0.29	14.25 ± 3.05
RF_1_YY	98.81 ± 1.61	13.8 ± 0.92*	114.98 ± 3.58*	84.03 ± 0.30	28.53 ± 0.23*	40.46 ± 1.28*
WRYY	94.44 ± 2.80	8.84 ± 0.13	91.13 ± 0.47	83.95 ± 1.70	23.96 ± 0.05	20.63 ± 1.21

Means of each trait were separated using t-test for independent samples. *Significant differences between hybrids and their respective weedy rice at P< 0.05 level. Data = Mean ± SE.

Under natural insect pressure, RF_1_ plants produced approximately 39.3-73.4% more panicles per plant and 15.9-29.7% greater 1000-grain weight compared to their respective weedy rice parents. Additionally, RF_1_MM, RF_1_TZ, and RF_1_YY produced 44.8%, 75.8%, and 26.2% more filled grains per panicle and 168.8%, 277.3% and 96.1% yield per plant, respectively, than their weedy rice counterparts (**Table 6**).

**Figure 9:**
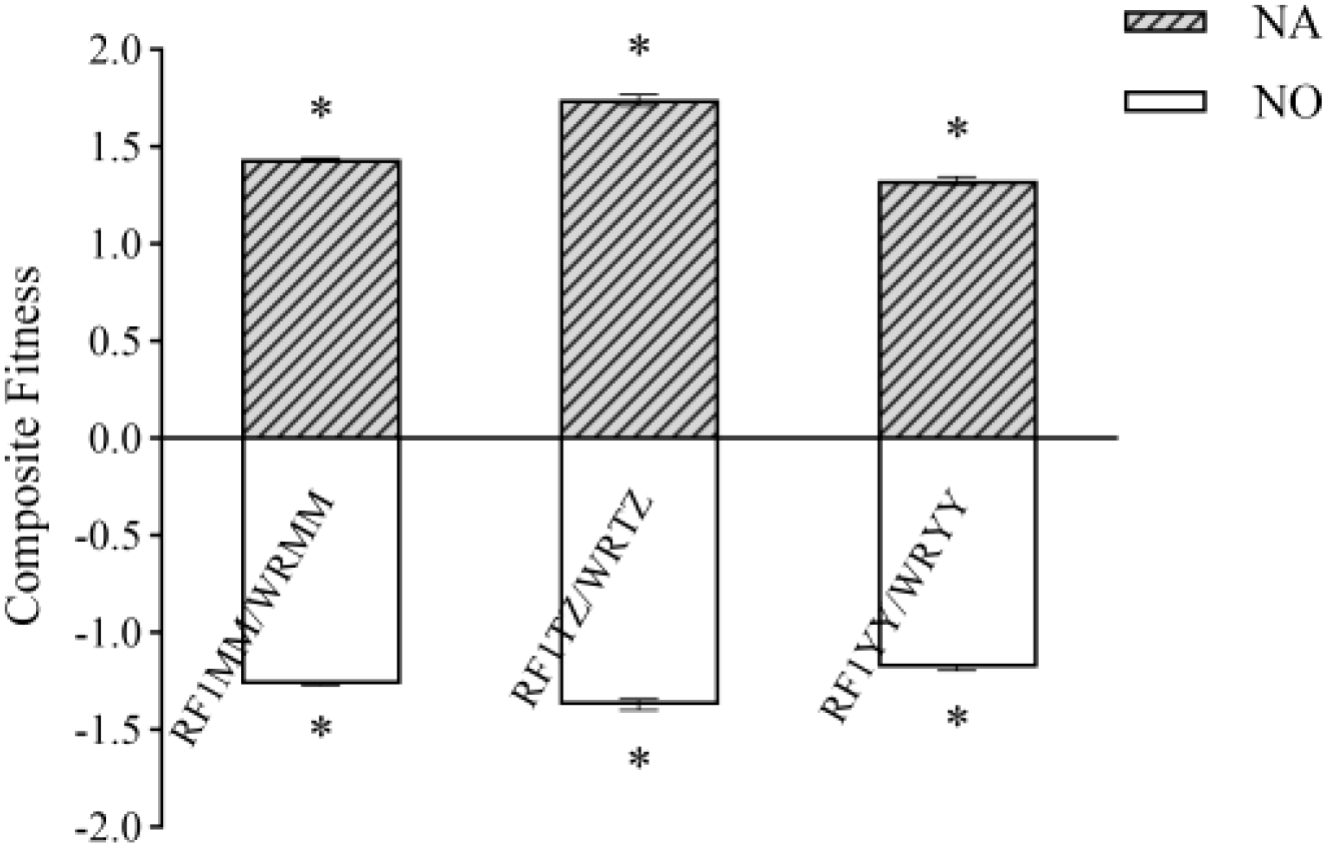
Fitness related traits of RF_1_ and its weedy rice paternal parent under natural or no insect pressure in monoculture planting. NA indicates under natural insect pressure; NO indicates under no insect pressure. (P < 0.05, error bar: SE, n=4). Means of composite fitness was separated using t-test for independent samples. * Significant differences between hybrids and their respective weedy rice at P < 0.05 level.

In general, RF_1_ plants had significantly greater composite fitness than their weedy rice counterparts, attributable to higher fecundity-related traits under both insect pressure or no insect pressure (**Figure 9**).

#### 3.2.4 Fitness of RF_1_ and F_1_

**Figure 10:**
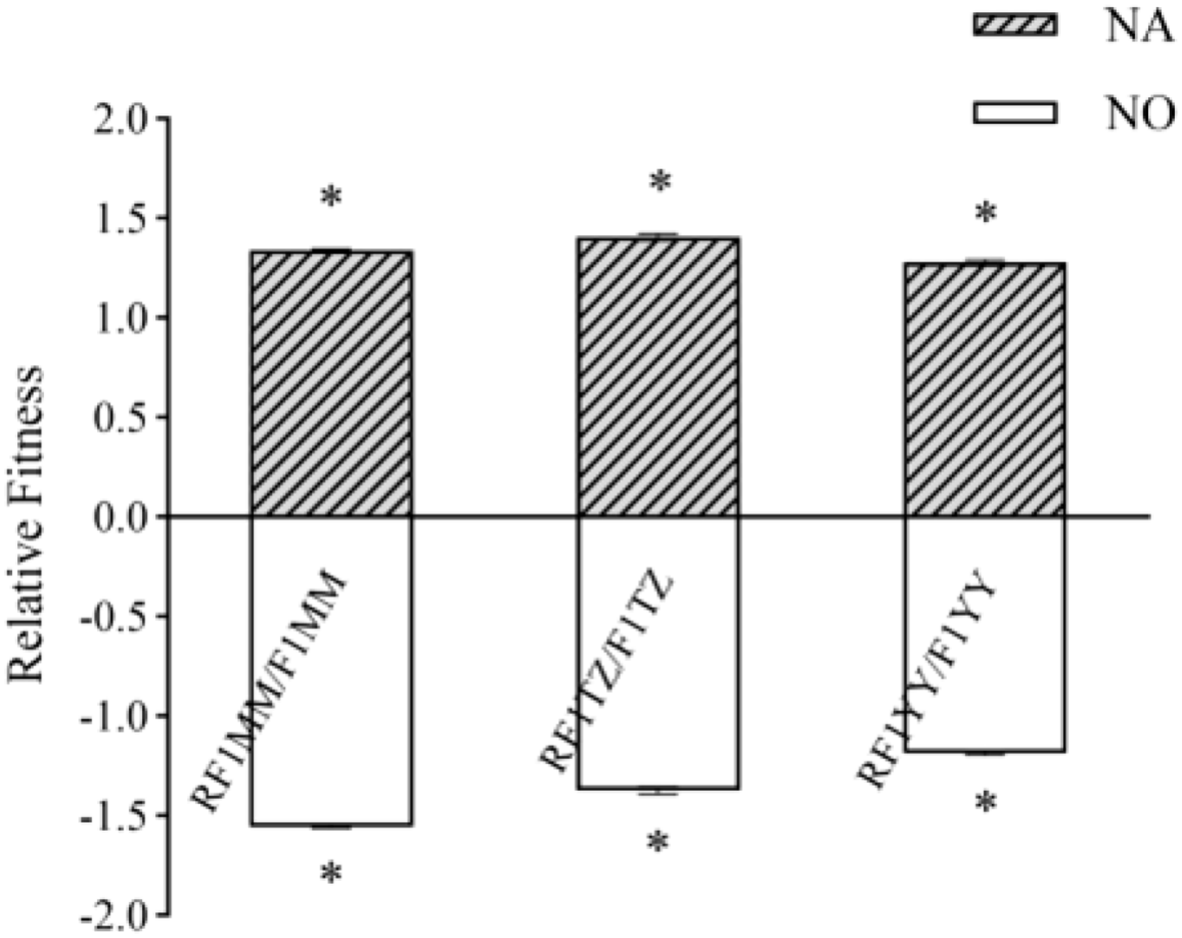
Fitness related traits of RF_1_ (T1c-19 as maternal parent) and F_1_ (T1c-19 as paternal parent) under natural or no insect pressure in monoculture planting. NA indicates under natural insect pressure; NO indicates under no insect pressure (P < 0.05, error bar: SE, n=4). Means of composite fitness was separated using t-test for independent samples. * Significant differences between RF_1_ hybrids and F_1_ hybrids at P <0.05 level.

When comparing the composite fitness of RF_1_ and F_1_, RF_1_ plants were significantly more fit than F_1_ plants under both under insect or no insect pressure (**Figure 10**).

### 3.3 Seed shattering of RF_1_ and weedy rice plants

**Figure 11:**
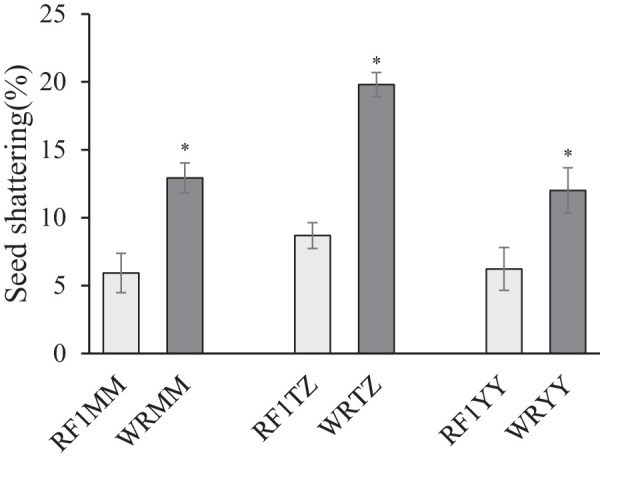
Seed shattering of weedy rice accessions and RF_1_ hybrids with T1c-19 (as the maternal plant) and weedy rice accessions (as paternal plants). WRMM, WRTZ and WRYY are weedy rice collected from Maoming, Taizhou and Yiyang, respectively. Data are mean ± SE (n=4). Means was separated using t-test for independent samples. * Significant differences between hybrids and their respective weedy rice at P< 0.05 level.

Among the three weedy rice accessions, WRTZ had the highest seed shattering (19.80%), while WRMM and WRYY had similar seed shattering of 12.93% and 12.00%, respectively. Compared to their weedy rice counterparts, the seed shattering of the three RF_1_ plants was significantly lower by 5.78-11.12%. There was no significant difference in seed shattering among the RF_1_ plants (**Figure 11**).

## 4 Discussion

### 4.1 Gene flow from weedy rice to cultivated rice

With respect to HR crops, gene flow does not differ whether the HR trait is introduced via genetic engineering or via conventional breeding techniques (Mallory-Smith and Olguin, 2011). Once the HR hybrids are produced through pollen-mediated gene flow from weedy rice to HR cultivated rice, the HR gene could further disperse to other weedy rice populations increasing the frequency of HR weedy rice.

In our preliminary research, we established a method for verifying F_1_ hybrids produced by reverse gene flow from weedy rice with red pericarp to cultivated rice (with white pericarp). This method used the 118 bp *Rc* gene of weedy rice as a marker. Sixty seeds collected from pollen recipient HR rice were grouped, ground to pass through a 60-mesh sieve, and tested for the *Rc* gene. This method proved effective, high-resolution, and cost-efficient for rapidly screening closely related cultivated rice hybrids produced by reverse gene flow.

In current research, reverse gene flow from three weedy rice accessions to T1c-19 differed: it was absent from WRTZ, while the frequencies were 0.0508% and 0.0808% from WRMM, and 0.0692% and 0.1008% from WRYY in alternating and adjacent cultivation designs, respectively. Thus, T1c-19 had the highest potential to evolve into HR weedy rice by accepting the pollen from WRYY, followed by WRMM. The probability of T1c-19 hybridizing with WRTZ was the lowest among the three weedy rice accessions.

Gene flow frequency varies depending on several factors, including reproductive compatibility between the rice cultivar and weedy rice biotype, sympatry, flowering synchrony, and length of overlapping flowering periods, plant height, experimental design, pollen source hectarage, and weather conditions (Zhang et al., 2003; Messeguer, 2003; Chen et al., 2004; Zhang et al., 2006; Shivrain et al., 2007, 2008; Zuo et al., 2011; Sun et al., 2015; Nam et al., 2019). Sexual reproduction of angiosperm involves a series of stages from pollen germination on the stigma to the development of the embryo and endosperm. Species that produce more seed under controlled pollination have a higher reproductive compatibility (Song et al., 2009). The differences of reverse gene flow among three weedy rice accessions likely relate to their sexual compatibility. In our previous experiment, seed setting was 39.8%, 10% and 6.3% when T1c-19 was manually crossed with WRYY, WRMM and WRTZ, respectively (unpublished data). The extent of sexual compatibility followed the same trend as the reverse gene flow frequency. Therefore, to avoid reverse gene flow, HR rice with lower sexual compatibility with local weedy rice should be cultivated.

Besides sexual compatibility, gene flow frequency is also influenced by pollen load (the number of pollen grains reaching the spikes of pollen recipients), which is related to overlapping flowering periods, coinciding daily flowering rhythms, and relative plant height of pollen donor and recipient (Shivrain et al., 2009; Zuo et al., 2011; Sun et al., 2015; Nam et al., 2019).

In this study, compared to T1c-19, WRMM was taller, and WRYY was similar, which likely facilitated the pollen of WRYY and WRMM in reaching the spikes of the shorter T1c-19, thereby increasing the chance for outcrossing. Conversely, WRTZ was shorter than T1c-19, likely decreasing the chance for outcrossing. This was the main reason for the absence of gene flow from T1c-19 to WRTZ.

In our previous research, in alternating and adjacent cultivations, the gene flow frequencies from T1c-19 to WRYY was 0.164 and 0.230%, and to WRTZ, 0.106 and 0.211%, respectively. However, no gene flow was detected with WRMM. The differences between direct and reverse gene flow results may be attributed to the shorter overlapping flowering periods between WRMM and T1c-19, and the much taller stature of WRMM compared to the pollen donor T1c-19. Additionally, the sexually compatibility of T1c-19 as maternal plant may differ with that as paternal plant, and variations during the two experimental periods, despite similar experimental designs, may have contributed to the results (Huang et al., 2015).

In conclusion, bidirectional gene flow between T1c-19 and three weedy rice accessions could produce HR weedy rice, complicating the evolution of HR weedy rice.

### 4.2 Effects of stacked transgenes on the fitness of F_1_ hybrids

The ecological risk of gene flow from cultivated crops to compatible weedy relatives is determined by the fitness of the F_1_ hybrids and subsequent generations (Song et al., 2009; 2011; Xia et al., 2011; Shukla et al., 2020). If the genes provide a selective advantage to crop-weed hybrids, greater fitness could lead to increased weediness (Chapman and Burke, 2006). The fitness of hybrids between insect or herbicide resistant crop and weedy relatives is affected by herbivore damage or herbicide pressure (Dechaine et al., 2009; Londo et al., 2010; Yang et al., 2011; 2012; 2015). Therefore, evaluating hybrids fitness under different selection pressures is essential for assessing gene flow risk for insect or herbicide resistant crops.

Compared to weedy rice, RF_1_ had significantly higher 1000-grain weight, yield per plant, and filled grain number per panicle, both with or without insect pressure. Thus, each plant of RF_1_ could produce 2–3 times of filled grains than weedy rice. It implied that RF_1_ hybrids could have a greater potential to produce more offspring, and the herbicide- and insect-resistant weedy rice populations could be more likely to establish population in field. Therefore, the higher composite fitness of RF_1_ means RF_1_ could cause potential ecological risk (Liu et al., 2016). Shivrain et al. (2009) reported that most hybrids produced by gene flow from weedy to cultivated rice yielded similar amounts of seed to that of the weedy parents. The differences in results between studies may be due to differences in parental genotype.

When comparing RF_1_ and F_1_, RF_1_MM and RF_1_TZ hybrids had 18.8-91.0% more panicles per plant, 26.4-31.3% more filled grains per panicle and 44.5-125.3% higher yield per plant than their F_1_ counterparts, regardless of insect pressure. RF_1_YY had 19.5% more panicles per plant under insect pressure and 12.3%-16.30% more filled grains per panicle, with a 30.7-35.0% higher yield per plant compared to RF_1_YY with or without insect pressure (Huang et al., 2019). In general, RF_1_ hybrids have a greater potential to produce more offspring, which could favor the establishment of herbicide- and insect-resistant weedy rice populations in field. Therefore, reverse gene flow may pose a significant ecological risk. This may be attributed to maternal effects in reciprocal crosses. Hybrids using cultivated crops as female parents result in weedy progenies with predominantly cultivated crop genes (De Wet and Harlan, 1975). Therefore, reverse gene flow from HR rice to weedy rice should receive careful attention. It is recommended that the reverse gene flow of HR rice, regardless of the method for cultivating it, should be evaluated before commercial release.

High seed shattering is a key trait for weedy rice, ensuring successful and efficient offspring dispersal (Wu et al., 2021). If weedy rice/cultivated rice hybrids have equal or higher seed shattering, they are more likely to escape harvesting and disperse seeds (Song et al., 2011). However, in this study, seed shattering characteristics of RF_1_ hybrids was partially suppressed compared to their weedy rice counterparts. The reduction in seed shattering could be unfavorable for RF_1_ hybrids, reducing their ability to avoid removal during grain harvest and persist in paddy fields. These findings are consistent with previous research on the seed shattering of F_1_ hybrids of weedy rice × T1c-19 (direct gene flow) (Huang et al., 2019), as well as studies on weedy rice × cultivated IMI-R rice (Shivrain et al., 2009; Serrat et al., 2013). The reason may be that F_1_ hybrids inherit the domestication trait of reduced seed shattering from cultivated rice. The major genes regulating seed shattering are *Sh4* and *qSH10* (Konishi et al., 2006; Li et al., 2006), Additionally, *qSH1*, *OsCPL1*, *OsXTH8* and *OsCel9* are important gene for seed shattering (Nunes et al., 2013). Other genes, including *SH5, SHAT1, SSH1, SH1, OSH15, GRF4, and NPC1*, are also involved in the loss of seed shattering (Wu et al., 2021). Further exploration of changes in these genes in hybrids compared to their parents is needed.

The lower seed shattering of RF_1_ hybrids implies that their seed would remain in the spike and thus be more likely removed during harvesting. This underscores the importance of using certificated cultivated rice seed to prevent the infestation of HR weedy rice mixed with cultivated rice seed (Serrat et al., 2013).

Seed dormancy, another key adaptive trait, regulates the timing of germination across seasons, enabling weedy rice to persist in agroecosystems (Gu et al., 2004; Pipatpongpinyo et al., 2020). Seed dormancy in rice interrelates to the weedy red pericarp color (Gu et al., 2005), and the pleiotropic locus most likely controls the dormancy and pigment traits by regulating ABA and flavonoid biosynthetic pathways, respectively (Gu et al., 2011). Furthermore, the natural genes controlling seed dormancy in weedy rice are involved in regulation of soil seedbank longevity (Pipatpongpinyo et al., 2020). Although the RF_1_ hybrids may display lower seed dormancy compared to its weedy rice counterparts, which could be unfavorable for their persistence in field, the accumulation of seed dormancy genes from weedy rice in HR transgenic cultivated rice could result in persistence of RF_1_ in rice tillage systems. Seed dormancy in RF_1_ was not investigated in this study and should be further researched.

In conclusion, gene flow via pollen from weedy rice to transgenic HR and IR rice can occur in natural field conditions, and HR hybrids produced by this reverse gene flow could survive in rice fields. Thus, measures, such as cultivating transgenic crop varieties which was cleistogamy, male sterility, chloroplast targeting and tandemly coupled mitigation genes, transgene excision, deleting transgene, or displayed lower genetic compatibility, or asynchronous flowering period with the associated weedy relatives, should be implemented (Madsen et al., 2002; Gressel and Valverde, 2009; Moon et al., 2010; Kwit et al., 2011; Clark and Maselko, 2020; Yang et al., 2021; Huang et al., 2023; Duan et al., 2023). Meanwhile, effective integrated weed management needs to be considered, such as using uncontaminated, certified seeds, applying the herbicide pre-emergence followed by post-emergence, clean machinery and crop rotation, must be taken to mitigate both reverse and direct gene flow (Roso et al., 2010; Sudianto et al., 2013; Avila et al., 2021; Unan et al., 2024).

Besides gene flow, herbicide selection drives the evolution of HR weedy rice (Qiu et al., 2020). Weedy rice can become acquired herbicide resistance through target-site resistance (TSR) or non–target site resistance (NTSR) when the Clearfield® (acetolactate synthase, ALS-inhibiting herbicide imidazolinone) or Provisia™ (acetyl-coenzyme A carboxylase, ACCase-inhibiting herbicide) rice is planted (Shivrain et al., 2010; Kaloumenos et al., 2013; Yean et al., 2021; Ruzmi et al., 2020, 2021; Gonzalez-Torralva and Norsworthy, 2023; Unan et al., 2024). HR weedy rice plants will be reduced effectiveness of the HR rice technology, and increased production costs and reduced yield (Avila et al., 2021; Ruzmi et al., 2020, 2021; Gonzalez-Torralva and Norsworthy, 2023; Unan et al., 2024).

HR weedy rice may evolve through bidirectional gene flow between rice and weedy rice, accompanied by repeated herbicide selection. Intensive adoption of HR rice technology, whether transgenic or non-transgenic, necessitates full attention for the management of HR weedy rice populations.

## Data Availability

The original contributions presented in the study are included in the article/**Supplementary Material**. Further inquiries can be directed to the corresponding author/s.
