# Supplementary material for: Gene flow from weedy rice to T1c-19 transgenic rice stacked with cry1C*/bar genes and fitness of F1 hybrids
Source: Front Plant Sci. 2025 Jul 22;16:1513367. doi: 10.3389/fpls.2025.1513367 (PMC12321772; doi:10.3389/fpls.2025.1513367)

The probability of *Rc* genes detected in DNA at different mixed ratios of WRYY and T1c-19 were showed in the table 1：

Number and the probability of *Rc* genes detected in DNA from 0.5 mL samples at different mixed ratios of WRYY and T1c-19.

| Seed mixed ratio  (WRYY : T1c-19) | The number of the *Rc* gene detected | The probability of the *Rc* gene detected |
| --- | --- | --- |
| 1:9 | 40 | 100% |
| 1:19 | 40 | 100% |
| 1:29 | 40 | 100% |
| 1:39 | 40 | 100% |
| 1:49 | 39 | 97.5% |
| 1:59 | 37 | 92.5% |
| 1:69 | 16 | 40% |
| 1:79 | 0 | 0 |
| 1:89 | 0 | 0 |
| 1:99 | 0 | 0 |

The results showed that the probability of detecting *Rc* gene at the 1:19 to 1:39 mixed ratios was 100%, and the probability of detecting *Rc* gene at 1:49 and 1: 59 was 97.5% and 92.5%, respectively. When the mixed ratio was 1:69, the probability of detecting *Rc* gene was only 40%, while *Rc* gene did not been detected in 1:79-1:99 mixed ratios. Therefore, 60 seeds collected from pollen recipients T1c-19 were identified as a group. Each group seeds were ground completely to passing through a 60-mesh sieve.


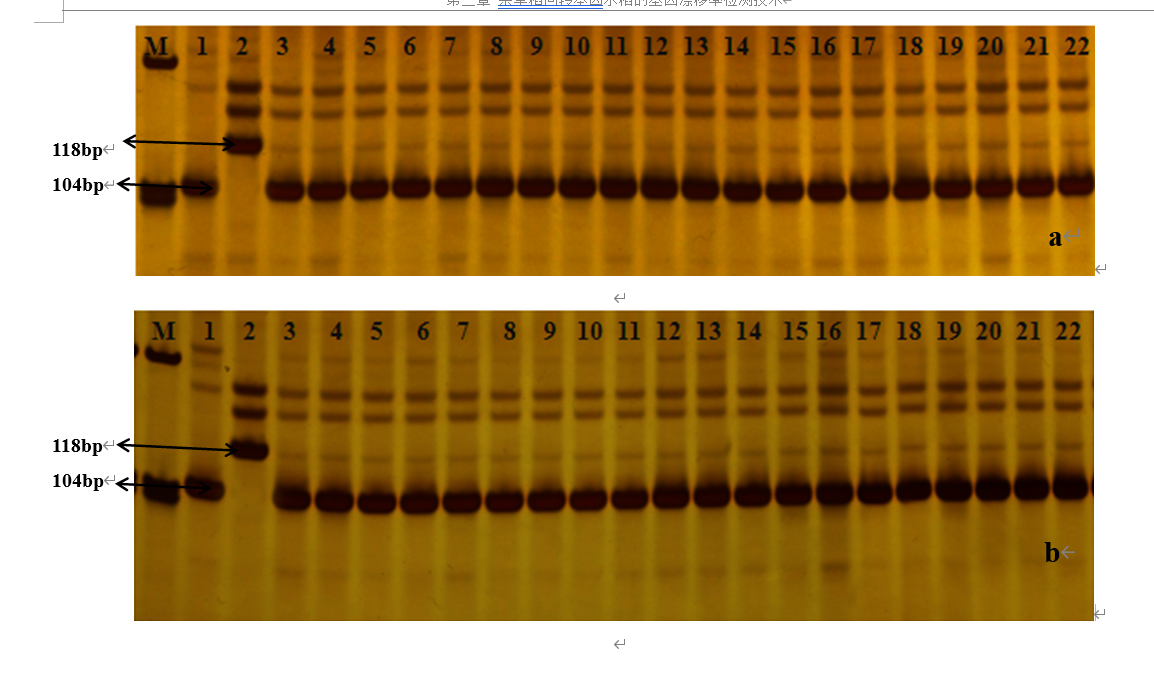

Supplement: Supplementary file 1 [file Table1.docx]
